# Comparative analysis of smoking cessation smartphone applications available in 2012 versus 2014

**DOI:** 10.1016/j.addbeh.2016.02.026

**Published:** 2016-07

**Authors:** Harveen Kaur Ubhi, Daniel Kotz, Susan Michie, Onno C.P. van Schayck, David Sheard, Abiram Selladurai, Robert West

**Affiliations:** a Cancer Research UK Health Behaviour Research Centre, University College London, London WC1E 6BT, UK; b Department of Family Medicine, CAPHRI School for Public Health and Primary Care, Maastricht University Medical Centre, Maastricht, The Netherlands; c Department of Clinical Effectiveness and Health Psychology, Centre for Outcome Research and Effectiveness, University College London, London WC1E 6BT, UK; d Institute of General Practice, Medical Faculty of the Heinrich-Heine, University Düsseldorf, Düsseldorf, Germany

**Keywords:** Smoking, Smoking cessation, Smartphone, Apps, Applications, Mobile, Behaviour change intervention, Behaviour change techniques, Taxonomy, BCTs, Engagement, Ease-of-use, Content analysis

## Abstract

**Background and aims:**

Smartphone applications (apps) offer a potentially cost-effective and a wide-reach aid to smoking cessation. In 2012, a content analysis of smoking cessation apps suggested that most apps did not adopt behaviour change techniques (BCTs), which according to previous research had suggested would promote higher success rates in quitting smoking. This study examined whether or not, this situation had changed by 2014 for free smoking cessation apps available in the Apple App Store. It also compared the use of engagement and ease-of-use features between the two time points.

**Methods:**

137 free apps available in the Apple App Sore in 2014 were coded using an established framework for the presence or absence of evidence-based BCTs, and engagement and ease-of-use features. The results from the 2014 data were compared with a similar exercise conducted on 83 free apps available in 2012.

**Results:**

BCTs supporting identity change, rewarding abstinence and advising on changing routines were less prevalent in 2014 as compared with 2012 (14.6% vs. 42.2%, 18.2% vs. 48.2%, and 17.5% vs. 24.1%, respectively). Advice on coping with cravings and advice on the use of stop-smoking medication were more prevalent in 2014 as compared with 2012 (27.7% vs. 20.5% and 14.6% vs 3.6%, respectively). The use of recognised engagement features was less common in 2014 than in 2012 (45.3% vs. 69.6%) while ease-of-use features remained very high (94.5% vs. 82.6%).

**Conclusion:**

There was little evidence of improvement in the use of evidence-based BCTs in free smoking cessation iPhone-based apps between 2012 and 2014.

## 1 Introduction

Smartphone applications (hereon referred to as ‘apps’) could provide support at a very low unit cost to those who wish to quit smoking. A previous content analysis assessed the extent to which smoking cessation apps available in the Apple App Store in 2012 contained behaviour change techniques (BCTs) that have been found to be effective in face-to-face support for smoking cessation (Ubhi, Michie, Kotz, et al., 2015). The study also examined the usage of engagement and ease-of-use features used within the apps (Ubhi et al., 2015). The study found that most of the apps available in the Apple App Store in 2012 focused on supporting identity change and rewarding abstinence (usually with praise) while a very small number of apps promoted the effective use of stop-smoking medication. The smoking cessation app market is rapidly evolving and it will be important to track the extent to which smoking cessation apps have become more evidence-based.

Mobile devices such as smartphones and tablets could transform the delivery of health promotion interventions. These devices have the capability to run apps, which are downloadable software products (Sherwood-Smith & Pritchard-Jones, 2012). Interventions delivered via smartphones and tablets can be used to deliver population-based interventions, which can be highly cost-effective. Interventions delivered via them are extremely customisable and can be used to provide 'real-time' interactive support as well as motivational information.

Among smokers in general, a high prevalence of smartphone ownership can be observed. A recent study reported that more than three-quarters of its sample of smokers in the US and the UK owned a smartphone and, of these, more than 80% had Internet access on their smartphones (Borrelli, Bartlett, Tooley, et al., 2015). Furthermore, the study found that a high prevalence of smokers had downloaded and used health apps alongside health websites in the recent past (Borrelli et al., 2015). The association between the high ownership of smartphones and a high interest in health apps indicates that app-based interventions can be used as a potential channel for smoking cessation. Due to an increasing interest in using apps to support quit attempts, it is important that smokers who are using these channels are provided with the appropriate advice to help them quit successfully.

Several content analyses have been conducted to evaluate the quality of smoking cessation apps that are available in the leading app stores (Abroms et al., 2011, Abroms et al., 2013, Choi et al., 2014, Jacobs et al., 2014, Hoeppner et al., 2015). Most of these analyses (Abroms et al., 2011, Abroms et al., 2013, Jacobs et al., 2014, Hoeppner et al., 2015) assessed smoking cessation apps in respect of their adherence to the US Public Health Service's Clinical Practice Guideline for Treating Tobacco Use and Dependence (Fiore, Jaén, Baker, et al., 2008). These analyses found that such adherence to the guideline was low (Abroms et al., 2011, Abroms et al., 2013, Jacobs et al., 2014) and that most of the available apps were not customised to users' needs (Hoeppner et al., 2015). Another recent content analysis evaluated smoking cessation apps based on the use of BCTs that were associated with improved success in face-to-face support for smoking cessation (Ubhi et al., 2015). BCTs are the ‘active ingredients’ of an intervention defined as ‘observable, replicable components of behaviour change interventions’ (Michie, Richardson, Johnston, et al., 2013). The results of that content analysis (Ubhi et al., 2015) extended previous findings (Abroms et al., 2011, Abroms et al., 2013, Choi et al., 2014, Jacobs et al., 2014, Hoeppner et al., 2015) in showing that smoking cessation apps lack evidence-based strategies to support smokers' quit attempts and, thus, available apps may not be providing the necessary help to motivated smokers.

For users to get exposed to BCTs that are delivered via smoking cessation apps, it is important that the apps are engaging and easy to use. App developers developing behaviour change apps need to incorporate features that could promote engagement (how users interact with different features present within the app) and usability by simplifying the user-interface (features that can make the app interface visually attractive, easy to use and intuitive). With apps that offer a service, it is relatively easy to engage app users by showing an immediate value or benefit. For example, some ‘entertainment’ apps such as travel apps allow travellers to upload and share photos with their friends via social networking websites while they are travelling. In this way, they can keep their friends up to date and can enjoy feedback about their ongoing travel experience. With regard to behaviour change apps such as those assisting smoking cessation, it is challenging to keep users active on the app because the benefits of using these apps may not be immediately apparent.

A mobile app store or a marketplace for apps (such as the Apple App Store and the Google Play Store) allows users to download apps on their smartphones. Both free apps and paid apps can be downloaded from such marketplaces. In the recent past, there has been an increase in the number of free apps (across all categories of apps, such as games, business, education, and health) (The History of App Pricing, And Why Most Apps Are Free, 2013). Between 2010 and 2012, the percentage of free apps varied between 80% and 84%; however, by the end of 2013, 90% of the apps in use were free (The History of App Pricing, And Why Most Apps Are Free, 2013). This may be attributed to the growing competition among app developers and the idea that most users want free content (The History of App Pricing, And Why Most Apps Are Free, 2013). The results of a pricing experiment led by developers using Flurry Analytics revealed that even charging $0.99 significantly reduced the demand for apps in the Apple App Store (The History of App Pricing, And Why Most Apps Are Free, 2013). When a user is searching for a smoking cessation app, there are often numerous free smoking cessation apps to choose from. Like paid apps, these free apps aid very similar tasks, such as goal setting (setting a quit date) and progress monitoring. Due to the changing consumer behaviour towards free apps, it is important to monitor how the smoking cessation app market, especially the free app market, has evolved in the last few years.

A previous content analysis of smoking cessation apps, conducted in 2012, investigated the content of paid and free apps available in the Apple App Store in terms of the use of BCTs, and engagement and ease-of-use features (Ubhi et al., 2015). The study found that some BCTs were quite well represented (e.g., supporting identity change and rewarding abstinence) while others received very little attention (particularly those advising on the use of stop-smoking medication). However, the overall use of BCTs was low in all the apps assessed. Engagement and ease-of-use features were widely applied, although the links between these features and the actual usage and effectiveness of the apps have not been demonstrated as yet.

The current study investigated the content of free smoking cessation apps available in the Apple App Store in December 2014 in terms of the use of BCTs, and engagement and ease-of-use features, and it compared the results with data from 2012 to assess the developments since that time. The current study only investigated free apps available in the Apple App Store. A recent review of apps found that paid apps contained just as many BCTs as free apps (Middelweerd, Mollee, van-der-Wal, et al., 2014). Moreover, the 2012 data did not find any association between the number of BCTs present and whether the app was free or not (Ubhi et al., 2015). In addition, there has been a shift in consumer behaviour towards downloading more free apps as compared with paid apps (The History of App Pricing, And Why Most Apps Are Free, 2013).

The main research question addressed was:

What changes took place in the inclusion of BCTs, and engagement and ease-of-use features in free smoking cessation apps available in the Apple App Store between September 2012 and December 2014?

## 2 Methods

### 2.1 Study design

**Fig. 1:**
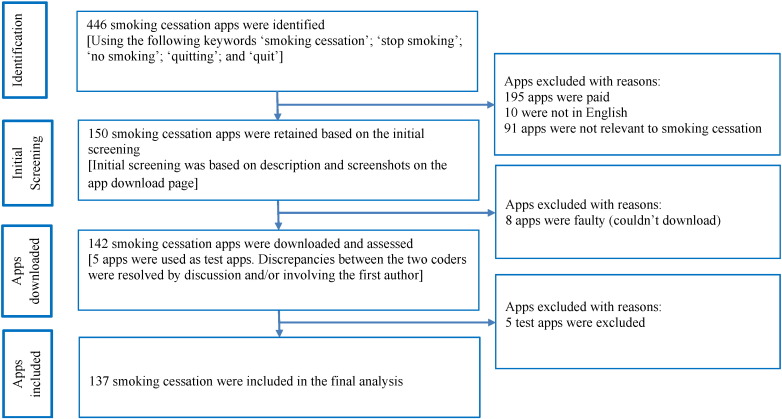
Selection of smoking cessation applications.

The Apple App Store market differs from country to country. The apps analysed in this study were searched and downloaded in the United Kingdom (UK) using a UK-based Apple App Store account (and therefore the list that appears in our search results may differ from other countries). The apps were searched in the Apple App Store on 4th December 2014 (www.apple.com/itunes) using the keywords ‘smoking cessation,’ ‘stop smoking’, ‘no smoking’, ‘quitting’, and ‘quit’. The study included those apps that were available for free, available in English and that purported to assist with smoking cessation (i.e., the primary purpose of the app was to aid smoking cessation). Of the total of 446 apps that were initially located, 142 met the inclusion criteria (Fig. 1) and were downloaded (see Supplementary file: Table A). The study used two independent coders who downloaded the 142 apps on the same day, thereby ensuring that they had the same version of the app. Each app was viewed on one occasion.

For the purposes of comparability between the 2012 and 2014 data, the 2012 data were reanalysed and included only free apps available in the Apple App Store at that time.

### 2.2 Coding of smoking cessation apps

**Fig. 2:**
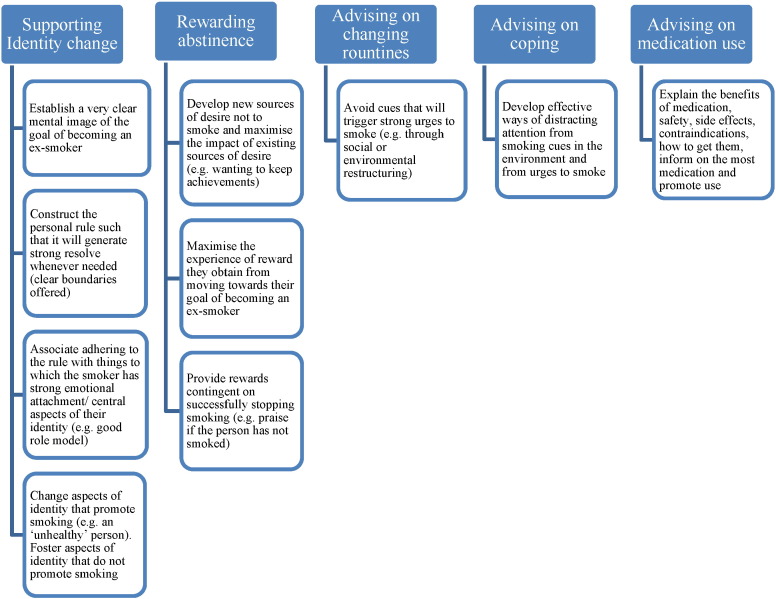
Framework for coding behaviour change techniques in smoking cessation smartphone applications.

The apps were coded for the inclusion of five BCTs that were expected to be effective in aiding smoking cessation (West, Walia, Hyder, et al., 2010). These were: 1) strengthening ex-smoker identity, 2) providing rewards (usually praise) contingent on stopping successfully, 3) advising on changing routines, 4) advising and assisting with ways of coping with urges to smoke, and 5) asking about and advising on the use of stop-smoking medication (Fig. 2). In addition, the apps were coded based on a set of 11 engagement features (Table 1) and a set of nine ease-of-use features (Table 2), as in the 2012 study.

Two coders independently assessed whether each of the five specific BCTs, and engagement and ease-of-use features was present or absent in each of the downloaded apps. There was no threshold for the number of times a particular BCT was used. One instance was enough for the BCT to be coded as present.

In the 2012 study, the two coders were given detailed information about the study but they were not given any further training to code the smoking cessation apps (Ubhi et al., 2015). However, in the current study, the two coders had undergone brief and structured training involving 8 h spread over 4 days. This training compromised of: 1) effectively using the smoking-specific taxonomy, and 2) identifying BCTs, and engagement and ease-of-use features present within smoking cessation apps. 

Prior to evaluating the full set of apps (n = 142) by the two independent coders, five apps were randomly selected and were used as test apps to assess the reliability between the two coders. The results from the test apps were discussed in order to understand any discrepancies between the coders. Disagreements were resolved by discussion and/or by involving the first author. The final analysis included the remaining 137 smoking cessation apps.

### 2.3 Statistical analysis

The statistical analysis was performed using IBM SPSS Statistics version 20.0. Inter-rater reliability was assessed by percentage agreement and by 'prevalence-adjusted bias-adjusted kappa'(PABAK) (Byrt, Bishop, & Carlin, 1993) for BCTs. For the interpretation of PABAK values, the following standard for strength of agreement was adopted: < 0.00 = poor, 0.00–0.20 = slight, 0.21–0.40 = fair, 0.41–0.60 = moderate, 0.61–0.80 = substantial and 0.81–1.00 = almost perfect agreement. A bias index was calculated by reflecting different propensities of the two coders to code a BCT as present. A prevalence index was calculated by indexing the combined likelihood of coding a feature as present. Intra-class correlation coefficients were calculated to assess the level of agreement between the coders in the scores for engagement and ease-of-use features. The prevalence estimates of BCTs were based on both the coders identifying a BCT in an app. As in the 2012 study, each of the five BCTs was considered separately, whereas for engagement and ease-of-use features, the percentage of possible features (11 and nine) identified in each app was computed to calculate a score from 0 to 1.0, and multiplying this score by 100 will give the proportion of features which were actually judged to be present within the apps. Thus, if an app used 5 engagement features, the score was [(5/11) ∗ 100] 45.5%. For the engagement and ease-of-use features, the scores of the two coders for each app were averaged. The current study did not conduct statistical tests to assess the statistical significance of differences in the prevalence of BCTs between 2012 and 2014 because the study was not seeking to generalise the result from a small sample to a much larger population, but rather it was characterising the whole population of interest (i.e. identifiable free apps available in the Apple App Store).

## 3 Results

In 2012, a total of 83 free smoking cessations apps available on the Apple app store were assessed while in 2014, 137 free smoking cessation apps available on the Apple app store were assessed (65% increase in the number of free smoking cessation apps). There were 110 new apps in 2014 while only 27 apps survived from 2012 to 2014. Out of 137 apps that were assessed in 2014, only 3 apps (2%) included all the five BCTs that could improve the chances of quitting smoking. Of these, the SF28 app (short for SmokeFree28) had the highest proportions of engagement (87%) and ease-of-use (100%) features present (see Supplementary file, Table A).

Table 3 shows that, from 2012 to 2014, there was a reduction in the percentage of apps that aimed to strengthen ex-smoker identity (n = 35, 42.2% vs. n = 20, 14.6%), provide contingent rewards (usually praise) on abstinence (n = 40, 48.2% vs. n = 25, 18.2%), and advise their users on changing routines (n = 20, 24.1% vs. n = 24, 17.5%). However, from 2012 to 2014, there was an increase in the percentage of apps that provided techniques to cope with cravings (n = 17, 20.5% vs. n = 38, 27.7%) and advised users on the use of stop-smoking medication (n = 3, 3.6% vs. n = 20, 14.6%).

In the 2012 data, the percentage agreement for BCTs ranged from 67.5% (advising on changing routines) to 95.2% (rewarding abstinence and advising on medication use). PABAK values ranged from 0.37 (supporting identity change and advising on changing routines) to 0.90 (advising on medication use) (p < 0.001 in all cases). In the 2014 data, the percentage agreement for BCTs had significantly increased and ranged from 85.4% (supporting identity change) to 92.7% (advising on coping with cravings). PABAK values ranged from 0.71 (supporting identity change) to 0.85 (advising on coping with cravings) (p < 0.001 in all cases). The intra-class correlation coefficients between the two coders for scores denoting the proportions of the set of engagement features, which were included, were 0.75 and 0.91 (p < 0.001), and of the set of ease-of-use features were 0.76 and 0.54 (p < 0.001) in 2012 and in 2014, respectively (Table 4).

The mean (SD) number of BCTs used in the apps was 1.37 (1.13) in 2012 as compared with 0.93 (1.29) in 2014. The use of specified engagement features that were used in the apps was 69.6% in 2012 and 45.3% in 2014. The use of specified ease-of-use features that were used in the apps was 82.6% in 2012 and 94.5% in 2014.

## 4 Discussion

The findings suggest that most free smoking cessation apps available in the Apple App Store in 2014 primarily focused on ease-of-use features and rarely used evidence-based BCTs. There was little evidence of improvement in the use of evidence-based BCTs in smoking cessation apps from 2012 to 2014.

The two BCTs (out of five) which were observed to be higher in the 2014 data as compared with the 2012 data were: 1) advising and assisting with ways of coping with urges to smoke; and 2) asking about and advising on the use of stop-smoking medication. The prevalence of the BCT asking about and advising on the use of stop-smoking medication had increased in the 2014 data as compared with the 2012 data (15% vs. 4%). In line with a recent content analysis of Android smoking cessation apps, the study found that a high proportion of apps discussed licensed pharmaceutical products (Hoeppner et al., 2015). Perhaps future smoking cessation apps could focus on providing support to their users on how to effectively use stop-smoking medication such as nicotine replacement therapy (NRT). For example, users could enter their choice of NRT (such as patches, gums, or nasal sprays) within the app and the app could then send its users with personalised messages in order to increase their adherence to the medication. Previous research suggests that smokers who buy NRT over-the-counter without any professional help have similar odds of stopping smoking as those who try to stop smoking without any aid (Kotz, Brown, & West, 2014), and this may be due to the inadequate use of NRT. Implementation of this feature within smoking cessation apps could significantly help app users who buy NRT over-the-counter without any professional help.

Previous content analyses of smoking cessation apps were conducted between June 2009 and August 2013 (Ubhi et al., 2015, Abroms et al., 2011, Abroms et al., 2013, Choi et al., 2014, Jacobs et al., 2014) and most of these analyses suggested that only a handful of apps recommended their users to use approved medication (Ubhi et al., 2015, Abroms et al., 2011, Abroms et al., 2013, Jacobs et al., 2014). The results from a recent content analysis (Hoeppner et al., 2015) and the results from the current study indicate a positive and significant change within smoking cessation apps over time. One could argue that perhaps the rise in the number of apps that advise app users to use stop-smoking medication could be attributed to the fact that pharmaceutical companies are developing smoking cessation apps with the objective of marketing their drugs rather than with the intent of improving the quality of smoking cessation apps. Besides this change (i.e., smoking cessation apps advising their users to use stop-smoking medication), most available apps used very little evidence-based content to support quit attempts. In 2014, approximately 55% (n = 75) of the apps assessed had no BCTs present while 31% (n = 43) had only up to two BCTs present. The results reveal that even the new apps that are being developed tend to lack the evidence-based content that is recommended to help smokers quit successfully. If such apps are developed with the ingredients that can improve the odds of quitting, then the interest with which smoking cessation apps are downloaded could provide a useful intervention at a low unit cost.

Another interesting observation in the apps assessed in the current study was that some of them had accompanying websites, books, DVDs and, e-coaching etc. While assessing these apps, it was observed that some of the free apps only revealed part of the information required for quitting and then directed users to buy additional material (such as books, DVDs or e-coaching) for added support. Some of the free apps had an ‘in-app’ purchase option, i.e., the users were requested to buy the full version of the app for extra support. As the conversion rates (defined as the percentage of users who transition from a free version of the product to a paid version) are fairly low (What's your conversion rate from trial to paid?, 2014), it is plausible to believe that very few users would actually purchase the additional material or make an ‘in-app’ purchase. Hence, the users of free apps may not be receiving end-to-end support, which is essential for a successful quit attempt.

The current study observed that the content of smoking cessation apps could be coded with high reliability in terms of the use of BCTs (almost perfect agreement was observed for four BCTs and a substantial agreement was observed for one BCT) as compared with the 2012 data where the reliability ranged from fair to high (Table 4). This could be due to the fact that the coders in the current study underwent a brief and structured training programme in using the smoking-specific taxonomy to identify BCTs, and engagement and ease-of-use features used within apps. This could have maximised the inter-coder reliability and confidence in using the taxonomy to evaluate apps. The inter-rater reliability for engagement and ease-of-use features remained high in both years (with the exception of ease-of-use features in 2014). This finding suggests that even without a training programme, it is easier to identify engagement and ease-of-use features as compared with identifying BCTs used within smoking cessation apps. One reason that could explain this finding is that BCTs are embedded within the text of a smoking cessation app and they could be challenging to recognise without appropriate training (i.e., BCTs can often be complex and can comprise several potentially interacting active components that can be difficult to identify). Although there are no online training programmes available to train coders on using the smoking-specific taxonomy, there are online training modules available that can train coders on the usage of generic taxonomies (Michie et al., 2013). Nonetheless, coders could still benefit from undertaking this training as few of the labels between the two taxonomies overlap.

The calculated engagement score in 2014 was lower as compared with the calculated engagement scores in 2012. These low scores suggest that most of the available apps are not engaging enough and hence may not be retaining their users. Thus, the overall success of the intervention might be affected. Research into health apps suggests that a substantial proportion of users stop using health apps soon after they have been downloaded (Krebs & Duncan, 2015). The most common reasons given by users for abandoning these apps are: 1) difficult to enter data in apps, 2) loss of interest (the apps no longer address their needs), and 3) data security and confidentiality (Krebs & Duncan, 2015). Previous research into health apps also reports a high drop-off rate among users as about one-third of the app users open health apps no more than once after downloading, while three-quarters of them open these apps fewer than ten times after downloading (Motivating Patients to Use Smartphone Health Apps — Consumer Health Information Corporation, 2011). The fact that the apps have such a high drop-off rate suggests that app developers should focus on including engagement features such as the use of gamification (Lister, West, Cannon, et al., 2014) [defined as the use of game design elements in non-game contexts (Deterding, Dixon, Khaled, et al., 2011)], which could increase engagement with the app. Moreover, to increase confidence among users using health apps which collect personal data, app developers can explicitly mention in the ‘terms and conditions’ and in the ‘privacy’ section of the app about the type of data that the app is going to collect and clearly state whether the users' data will be shared with third parties. On the other hand, the ease-of-use score remained high in 2014 and in 2012. This trend reflects the fact that app developers are paying more attention to creating apps that are easy to use.

The findings of this study should be interpreted in the context of some key limitations. Firstly, the apps in the study were viewed on only one occasion. Some of the apps that were evaluated required their users to login multiple times in order to get exposed to all the content and features that were available. For a more comprehensive assessment, each app would have to be used just as a quitter would use it (for a few days at least). As these apps were assessed on only one occasion, there is a possibility that the prevalence estimates of BCTs are under-reported in this paper. It is indeed a challenging task to decide whether or not these apps should be revisited at another time in order to evaluate the full set of features, which may not have been uncovered during the app's first-time use. This requires establishing consensus among behavioural scientists on how to code tunnelled digital interventions effectively. The coders in our present study were free to browse the app for as long as they needed until the available content was meticulously explored. Secondly, some of the apps assessed in the study had accompanying websites, books, DVDs or e-coaching. The credibility of this additional material in terms of the use of BCTs and other features was not assessed by the coders. This too adds another layer of complexity in terms of evaluating these apps. Thirdly, only apps available in the Apple App Store were evaluated. Most of the apps available in the leading app stores follow similar design patterns for both Apple's iPhones and Google's Android phones. Therefore, it seems unlikely that the inclusion of Android apps will produce substantially different results in terms of the use of BCTs, and engagement and ease-of-use features. Fourthly, only free smoking cessation apps were evaluated. Due to a high interest in free apps among app users, it is a useful exercise to evaluate free smoking cessation apps. In addition, previous reviews have found no difference between the number of BCTs present within an app and whether the app was free or not. Thus, it is plausible to believe that including paid apps in the review would not have significantly altered the results.

In summary, the findings suggest that most free smoking cessation apps available in the Apple App Store primarily focused on ease-of-use features and also, to some extent, on engagement features, but they rarely focused on evidence-based BCTs that are known to improve the success of quit attempts. There was little evidence of improvement in the use of evidence-based BCTs in smoking cessation apps from 2012 to 2014.

The following are the supplementary data related to this article.

Table A Names of the 137 smoking cessation apps that were reviewed in terms of BCTs, and the proportion of engagement and ease-of-use features used within these apps (the apps in table are arranged in descending order of the BCTs present).

## Statement of competing interests

Harveen Kaur Ubhi, Susan Michie, David Sheard and Abiram Selladurai declare that they have no conflict of interest. Robert West and Onno C.P. van Schayck have undertaken research and consultancy for companies that develop and manufacture smoking cessation medications. Daniel Kotz received an unrestricted grant from Pfizer for a smoking cessation trial. All procedures were conducted in accordance with the ethical standards of the responsible committee on human experimentation (institutional and national) and with the Helsinki Declaration of 1975, as revised in 2000.

## Funding and acknowledgements

This study was funded by the National Centre for Smoking Cessation and Training (NCSCT). The funders played no role in the design, conduct or analysis of the study, nor in the interpretation and reporting of the study findings. The researchers were independent from the funders.

Harveen Kaur Ubhi had full access to all of the data (including statistical reports and tables) in the study and can take responsibility for the integrity of the data and the accuracy of the data analysis.

Robert West is funded by Cancer Research UK and is a member of the UK Centre for Tobacco and Alcohol Studies. Daniel Kotz is funded by the Ministry for Innovation, Science and Research of the German Federal State of North Rhine-Westphalia (“NRW-Rückkehrprogramm”).

## Figures and Tables

**Table 1:** App features that could promote engagement.

	Feature	Brief description
1	Personas and personification	Establish a ‘rapport’ between the smoker and personification of the app (e.g., by creating a visual sense of the team)
2	Transparency and realistic expectations	Set up clear expectations concerning how the app will be used early on
3	Shaping	Keep demands of the smoker to a minimum
4	Instant feedback/gratification/gamification (scoreboards, points, badges, leader-boards, achievements, assignments etc.)	Engage users by providing instant feedback loops (provide user progression statistics). Always provide users with a rewarding experience when they visit the app (rewards motivate people for more rewards)
5	Visual cues and dashboards	Where possible use images (photos, graphics, or videos) to convey information
6	Design for curiosity	Present new information each time the app is accessed
7	Personalisation	Promote engagement by using text messaging and emails
8	Autonomy	Give control, choice and personal relevance by asking questions
9	Personalised recommendations	Make the app as interactive as possible — ask relevant questions, tailored feedback, videos, audio, gallery, emails, text messaging, etc.
10	App's design and user interface	The app must look professional
11	Sequencing and design for reducing each session time	Structure sections (break complex tasks into small steps) and keep login sessions brief (each session should not take more than 5 min of the users' time)

**Table 2:** App features that could enhance ease-of-use.

	Feature	Brief description
1	Pattern recognition	Make use of the app as habitual as possible in terms of the location of different elements
2	Aesthetics	Keep main pages as simple and visually appealing as possible but encourage and make it easy to use
3	Minimum text	Keep text as brief as possible
4	Text formatting	Try to avoid grouping more than two sentences together, use plenty of headings, keep paragraphs short and use bulleted lists and highlight key terms
5	Page names	Navigation must be consistent and straightforward. Every page needs a name, the name needs to be in the right place (in the visual hierarchy of the page, the page name should appear to be framing the content that is unique to this page), the name needs to be prominent (combination of size, colour and typeface), the name needs to match with what user clicked
6	Easy-to-read	Reading level to age 14
7	Layout	Layout pages to avoid scrolling on the most popular screen resolution
8	Clear and consistent language	Keep consistency throughout with regard to layout and grammar
9	Font size	Avoid small text

**Table 3:** Prevalence estimates for specific behaviour change techniques (BCTs) in free smoking cessation applications in 2012 and 2014.

BCT	Prevalence estimate	Prevalence estimate
	2012 (N = 83)	2014 (N = 137)
Supporting identity change	42.2% (n = 35)	14.6% (n = 20)
Rewarding abstinence	48.2% (n = 40)	18.2% (n = 25)
Advising on changing routines	24.1% (n = 20)	17.5% (n = 24)
Advising on coping with cravings	20.5% (n = 17)	27.7% (n = 38)
Advising on medication use	3.6% (n = 3)	14.6% (n = 20)

**Table 4:** Percent agreement and PABAKs for specific behaviour change techniques (BCTs) that are found to be associated with higher success rates for smoking cessation in 2012 and 2014.

BCT	Percentage agreement	Prevalence index	Bias index	PABAK	Percentage agreement	Prevalence index	Bias index	PABAK
	2012				2014			
Supporting identity change	68.7	0.16	0.14	0.37	85.4	− 0.56	− 0.12	0.71
Rewarding abstinence	95.2	0.14	0.08	0.64	89.8	− 0.53	− 0.07	0.80
Advising on changing routines	67.5	− 0.23	0.29	0.37	90.5	− 0.55	− 0.04	0.81
Advising on coping with cravings	74.7	− 0.34	0.25	0.49	92.7	− 0.37	− 0.03	0.85
Advising on medication use	95.2	− 0.88	0.05	0.90	91.2	− 0.62	0.04	0.82

PABAK = prevalence-adjusted bias-adjusted kappa.
