# Supplementary material for: Comparative analysis of smoking cessation smartphone applications available in 2012 versus 2014
Source: Addict Behav. 2016 Jul;58:175–81. doi: 10.1016/j.addbeh.2016.02.026 (PMC4821061; doi:10.1016/j.addbeh.2016.02.026)
Supplement: Table A — Names of the 137 smoking cessation apps that were reviewed in terms of BCTs, and the proportion of engagement and ease-of-use features used within these apps (the apps in table are arranged in descending order of the BCTs present). [file mmc1.docx]

**Supplementary Section**

Table A: Names of the 137 smoking cessation apps that were reviewed in terms of the use of BCTs, and the proportion of engagement and ease-of use features used within these apps (the apps in the table below are arranged in descending order of the number of BCTs present).

| **Name of the Smoking Cessation App** | **BCT 1: Supporting identity change** | **BCT 2:**  **Rewarding abstinence** | **BCT 3:**  **Advising on changing routines** | **BCT 4:**  **Advising on changing routines** | **BCT 5:**  **Advising on medication use** | **Total number of BCTs present**  **(out of 5)** | **Engagement Features** | **Ease-of-use Features** |
| --- | --- | --- | --- | --- | --- | --- | --- | --- |
| **Apps with all the five BCTs present (3/137 apps)** | | | | | | |  |  |
| SF28 (SmokeFree28) | ✓ | ✓ | ✓ | ✓ | ✓ | 5 | 87% | 100% |
| HPB I Quit | ✓ | ✓ | ✓ | ✓ | ✓ | 5 | 64% | 84% |
| Stop Smoking Now! | ✓ | ✓ | ✓ | ✓ | ✓ | 5 | 36% | 89% |
| **Apps with any four BCTs present (5/137 apps)** | | | | | | |  |  |
| Crush The Crave | X | ✓ | ✓ | ✓ | ✓ | 4 | 82% | 95% |
| KickTheButt | ✓ | X | ✓ | ✓ | ✓ | 4 | 32% | 62% |
| NCI QuitPal | X | ✓ | ✓ | ✓ | ✓ | 4 | 78% | 100% |
| Quit And Be Free | ✓ | ✓ | ✓ | ✓ | X | 4 | 36% | 89% |
| QuitGuide | ✓ | X | ✓ | ✓ | ✓ | 4 | 32% | 78% |
| **Apps with any three BCTs present (11/137 apps)** | | | | | | |  |  |
| Give Up Smoking - Learn How To Get The Right Help To Quit Smoking | X | X | ✓ | ✓ | ✓ | 3 | 14% | 50% |
| LIVESTRONG MyQuit Coach - Dare To Quit Smoking | ✓ | ✓ | X | ✓ | X | 3 | 78% | 100% |
| Nicorette ActiveStop | X | X | ✓ | ✓ | ✓ | 3 | 82% | 100% |
| Nicotinelle Unifiltered | X | ✓ | X | ✓ | ✓ | 3 | 73% | 100% |
| Quit For You - Quit For Two | ✓ | ✓ | X | ✓ | X | 3 | 64% | 100% |
| R2Q Ready To Quit Smoking | ✓ | X | ✓ | ✓ | X | 3 | 50% | 100% |
| SmokeFree Baby - Stop Smoking | ✓ | X | X | ✓ | ✓ | 3 | 78% | 89% |
| smokeFree Lite | ✓ | X | ✓ | ✓ | X | 3 | 41% | 100% |
| SmokeLess Quit | ✓ | X | ✓ | ✓ | X | 3 | 55% | 100% |
| Stay Quit Coach | ✓ | X | X | ✓ | ✓ | 3 | 73% | 84% |
| Tobacco Free Teens | ✓ | X | ✓ | ✓ | X | 3 | 60% | 100% |
| **Apps with any two BCTs present (16/137) apps** | | | | | | |  |  |
| Cloud9WLDAS | X | X | X | ✓ | ✓ | 2 | 45% | 100% |
| FREE Stop Smoking Cigarettes Now Quit Smokes Forever Tracker | ✓ | X | X | ✓ | X | 2 | 60% | 100% |
| Kick It! | ✓ | X | X | ✓ | X | 2 | 55% | 100% |
| Mental Workout | X | X | X | ✓ | ✓ | 2 | 69% | 100% |
| NHS Stop Smoking | X | X | X | ✓ | ✓ | 2 | 50% | 100% |
| Quit For Life | X | X | ✓ | ✓ | X | 2 | 55% | 100% |
| Quit Now: My QuitBuddy | X | X | ✓ | ✓ | X | 2 | 78% | 100% |
| QuitNow | X | X | ✓ | ✓ | X | 2 | 50% | 89% |
| QuitSTART | X | ✓ | X | ✓ | X | 2 | 64% | 100% |
| San Francisco Stop Smoking | X | X | ✓ | ✓ | X | 2 | 73% | 84% |
| Smokefree | X | X | ✓ | ✓ | X | 2 | 82% | 100% |
| Stop Smoking - Hypnosis | X | X | ✓ | ✓ | X | 2 | 23% | 78% |
| Stop-Tobacco | X | X | ✓ | ✓ | X | 2 | 55% | 84% |
| uQuitSmoking | X | X | X | ✓ | ✓ | 2 | 36% | 89% |
| The Best No Smoking App | X | X | ✓ | ✓ | X | 0 | 45% | 89% |
| Wero | X | X | ✓ | ✓ | X | 2 | 60% | 100% |
| **Apps with any one BCTs present (27/137 apps)** | | | | | | |  |  |
| 3-2-1 Quit Smoking Now! | X | X | X | X | ✓ | 1 | 50% | 84% |
| BetterYou Hypnosis - The Ultimate Self Improvement App (NLP) | X | X | X | ✓ | X | 1 | 18% | 95% |
| JustQuit - Quit Smoking App For A Healthy Smokefree Life | X | ✓ | X | X | X | 1 | 41% | 95% |
| Kwit - Quit Smoking Is A Game | X | ✓ | X | X | X | 1 | 45% | 95% |
| Life Coach - FREE Self Improvement, Personal Development and Hypnotherapy | ✓ | X | X | X | X | 1 | 27% | 100% |
| MyQuitSmokingCoach: Europe's No. 1 Quit Smoking APP | X | ✓ | X | X | X | 1 | 64% | 84% |
| MyTimeToStart | X | X | X | X | ✓ | 1 | 50% | 100% |
| No Smoke Coach | X | ✓ | X | X | X | 1 | 73% | 89% |
| NoSmokingLife | X | X | ✓ | X | X | 1 | 45% | 100% |
| Puff Away-Stop Smoking Today | X | ✓ | X | X | X | 1 | 50% | 100% |
| Quiit Smoking | ✓ | X | X | X | X | 1 | 41% | 95% |
| Quit & Get Fit | X | X | X | ✓ | X | 1 | 64% | 100% |
| Quit For Health Lite | X | ✓ | X | X | X | 1 | 36% | 100% |
| Quit Pro: Your Smoking Cessation Coach | X | ✓ | X | X | X | 1 | 45% | 100% |
| Quit Smoking - QuitNow! | X | ✓ | X | X | X | 1 | 50% | 100% |
| Quit Smoking And Stay Healthy Free | X | ✓ | X | X | X | 1 | 45% | 95% |
| Quit Smoking Hypnosis - FREE Guided Meditation and the Best Hypnotherapist | ✓ | X | X | X | X | 1 | 18% | 100% |
| Quit Smoking Together | X | ✓ | X | X | X | 1 | 59% | 100% |
| QuitCharge - Stop Smoking | X | X | X | ✓ | X | 1 | 69% | 100% |
| Quitting Buddy - The Stop Smoking App With A Difference | X | ✓ | X | X | X | 1 | 78% | 100% |
| Smoke FREE - Finally Non Smoking | X | ✓ | X | X | X | 1 | 41% | 89% |
| Smokefree - Quit Smoking Now! | X | ✓ | X | X | X | 1 | 36% | 95% |
| Smoking Cessation Decision Aid | X | X | X | X | ✓ | 1 | 69% | 84% |
| Stop Smoking! | ✓ | X | X | X | X | 1 | 14% | 89% |
| StopSmoke | X | X | X | X | ✓ | 1 | 41% | 84% |
| Streaks For Small Starts - Create Habits With Simple, Daily Acitivites | X | ✓ | X | X | X | 1 | 41% | 100% |
| YoPuedo - Gives You The Help You Need To Quit Smoking Once And For All | X | ✓ | X | X | X | 1 | 41% | 100% |
| **Apps with no BCTs present (75/137 apps)** | | | | | | |  |  |
| Quit That! - Track How Long Since You Stopped Your Bad Habits And Addictions | X | X | X | X | X | 0 | 36% | 100% |
| Affirmation Alarm | X | X | X | X | X | 0 | 32% | 95% |
| Antifumo | X | X | X | X | X | 0 | 27% | 78% |
| BeFree! | X | X | X | X | X | 0 | 32% | 100% |
| Call It Quits | X | X | X | X | X | 0 | 55% | 100% |
| Can I Smoke? | X | X | X | X | X | 0 | 36% | 100% |
| CigaretteTracker | X | X | X | X | X | 0 | 36% | 100% |
| CIGGY: The Ill Fated Terribly Doomed Love Affair | X | X | X | X | X | 0 | 41% | 89% |
| Crave Out! | X | X | X | X | X | 0 | 23% | 95% |
| Craving To Quit | X | X | X | X | X | 0 | 78% | 100% |
| Done Smoking | X | X | X | X | X | 0 | 41% | 95% |
| Gasper | X | X | X | X | X | 0 | 32% | 95% |
| Good Resolutions | X | X | X | X | X | 0 | 18% | 89% |
| Gotta Smoke? | X | X | X | X | X | 0 | 36% | 100% |
| iQSmoking | X | X | X | X | X | 0 | 55% | 100% |
| iQuit | X | X | X | X | X | 0 | 60% | 100% |
| iQuit Counter | X | X | X | X | X | 0 | 36% | 95% |
| iQuit for iOS | X | X | X | X | X | 0 | 36% | 100% |
| iQuitSmoking! | X | X | X | X | X | 0 | 50% | 100% |
| iSmoke Too Much - Beware & Aware | X | X | X | X | X | 0 | 36% | 100% |
| Kick The Habit: Quit Smoking | X | X | X | X | X | 0 | 41% | 100% |
| Kickit 2 - Quit Smoking, Incentivised | X | X | X | X | X | 0 | 32% | 89% |
| LiveWell Richmond | X | X | X | X | X | 0 | 41% | 100% |
| Motivation by Sgt Heartman | X | X | X | X | X | 0 | 14% | 95% |
| Motivator - Stop Smoking With Your Own Personal Motivator! | X | X | X | X | X | 0 | 50% | 84% |
| My Hypnosis Stop Smoking Lite Version | X | X | X | X | X | 0 | 5% | 100% |
| My Last Cigaratte FREE - Stop Smoking Stay Quit | X | X | X | X | X | 0 | 45% | 95% |
| My Quit Smoking Math from TheQuitSmokingGuy.com | X | X | X | X | X | 0 | 36% | 100% |
| MyQuitTime Free | X | X | X | X | X | 0 | 36% | 100% |
| No Smoking (Diary) | X | X | X | X | X | 0 | 27% | 95% |
| No Smoking Game | X | X | X | X | X | 0 | 23% | 95% |
| NoGo | X | X | X | X | X | 0 | 41% | 100% |
| Non Smoking Counter | X | X | X | X | X | 0 | 45% | 89% |
| OAP Yourself | X | X | X | X | X | 0 | 36% | 100% |
| Phone Smoker - Stop Smoking | X | X | X | X | X | 0 | 36% | 100% |
| Quit It Lite - Stop Smoking Now | X | X | X | X | X | 0 | 36% | 95% |
| Quit Smoking Buddy - Stop Smoking Coach! | X | X | X | X | X | 0 | 64% | 100% |
| Quit Smoking Free | X | X | X | X | X | 0 | 32% | 89% |
| Quit Smoking Helper | X | X | X | X | X | 0 | 45% | 84% |
| Quit Smoking Hypnosis by Mindifi - Stop Addiction Now | X | X | X | X | X | 0 | 27% | 100% |
| Quit Smoking: Learn To Stop Smoking Today! | X | X | X | X | X | 0 | 45% | 100% |
| Quitbit - Motivation to Quit Smoking | X | X | X | X | X | 0 | 45% | 95% |
| QuitCounter | X | X | X | X | X | 0 | 41% | 95% |
| QuitNow - No Smoking With A TWEET | X | X | X | X | X | 0 | 45% | 100% |
| Shocking Smoking Facts | X | X | X | X | X | 0 | 27% | 78% |
| Since - Date and Time Counter | X | X | X | X | X | 0 | 36% | 100% |
| Since iQuit | X | X | X | X | X | 0 | 36% | 100% |
| Smoke Diary | X | X | X | X | X | 0 | 50% | 100% |
| Smoke Free - Quit Smoking Now And Stop For Good | X | X | X | X | X | 0 | 69% | 100% |
| Smoke Tracker | X | X | X | X | X | 0 | 36% | 100% |
| Smoke-Alarm | X | X | X | X | X | 0 | 60% | 100% |
| Smoked BBOX | X | X | X | X | X | 0 | 23% | 100% |
| SmokErOut - The Smokers and Quitting Smoking Game | X | X | X | X | X | 0 | 32% | 100% |
| SmokerSafe | X | X | X | X | X | 0 | 46% | 95% |
| Smoking Cessation | X | X | X | X | X | 0 | 69% | 100% |
| Smoking Cool Down | X | X | X | X | X | 0 | 23% | 95% |
| Smoking Management | X | X | X | X | X | 0 | 36% | 95% |
| Smokx | X | X | X | X | X | 0 | 23% | 61% |
| So You Think You Can Quit For iPhone/iPad | X | X | X | X | X | 0 | 41% | 100% |
| Stop Smoking - Mindfulness Meditation App To Cessation Smoking | X | X | X | X | X | 0 | 14% | 100% |
| Stop Smoking App | X | X | X | X | X | 0 | 46% | 78% |
| Stop Smoking Cessation | X | X | X | X | X | 0 | 64% | 89% |
| Stop Smoking Instantly With Chinese Massage Points - FREE | X | X | X | X | X | 0 | 64% | 100% |
| Stop Smoking. Subliminal Hypnotherapy Free | X | X | X | X | X | 0 | 18% | 62% |
| Stop! | X | X | X | X | X | 0 | 23% | 89% |
| Stub It Out | X | X | X | X | X | 2 | 46% | 100% |
| The Painless Stop Smoking Cure | X | X | X | X | X | 0 | 64% | 95% |
| Tic uR Life - Smoke-Free! | X | X | X | X | X | 0 | 55% | 100% |
| Tobacco Free Family | X | X | X | X | X | 0 | 55% | 100% |
| Tobacco Quit and Save | X | X | X | X | X | 0 | 36% | 100% |
| Trigger Monitor - The Breaking Bad Habit Program | X | X | X | X | X | 0 | 45% | 100% |
| Unsmoke | X | X | X | X | X | 0 | 27% | 100% |
| YouCanQuit | X | X | X | X | X | 0 | 46% | 100% |
| ACU Low Health Literacy Nicotine Addiction Test | X | X | X | X | X | 0 | 36% | 100% |
| The Kerry Gaynor Method | X | X | X | X | X | 0 | 41% | 100% |
